# Exploring Health Priorities in the Barbershop: A Modified Delphi Study of Barbers’ and Clients’ Views on What is Important and What is Feasible

**DOI:** 10.1007/s10900-026-01584-9

**Published:** 2026-05-29

**Authors:** Kaylyn A. Garcia, Kalyn Prothro, Trishna Patel, Micheal Priester, Terry Woods, Guillermo Wippold

**Affiliations:** 1 Department of Psychology, University of South Carolina, Columbia, SC USA; 2 Department of Nursing, University of South Carolina, Columbia, SC USA; 3 Main Attraction Barbershop, Sumter, SC USA

**Keywords:** Health promotion, Barbershops, Black or African American, Men, Delphi

## Abstract

Barbershops are a place of cultural significance for many Black men and have been the site of successful health promotion programming; yet, efforts conducted in barbershops should recognize the contextual and organizational constraints of the site. The present study used a modified Delphi poll to identify health topics that were important among Black men and feasible to address in the barbershop according to barbers (Round 1) and clients (Round 2). A total of 63 individuals participated in this study (n = 29 barbers and n = 34 clients). Barbers were presented with 54 health topics and were asked to rate the importance and feasibility of addressing each health topic among Black men in the barbershop. Results were then plotted in a Go-Zone plot with four quadrants—(Go-Zone 1) high importance and feasibility;(Go-Zone2)low importance and high feasibility;(Go-Zone3) low importance and feasibility ;and(Go-Zone4)high importance and low feasibility. Results in Go-Zones 1, 2, and 4 were then presented to clients. Results were then plotted again and t-tests were performed to examine differences between barbers and clients. Final results visually display the importance/feasibility of each health concern according to barbers and clients. Additionally, t-test analyses indicate that barbers view health concerns as more important/feasible than their clients. The results of this study have important implications for the contextually-aligned design and implementation of health promotion programming in barbershops.

## Introduction

### Health Among Black Men

Black men in the United States (U.S.) have among the lowest life expectancy of any racial/ethnic-gender group in the United States. The life expectancy of Black men in the U.S. is 67.6 years of age, which is lower than that of Asian men (81.2 years), Hispanic men (74.6 years), White men (74.0 years), and all women (79.3 years) [1]. National mortality surveillance data indicates that the top five causes of death among Black men (i.e., heart disease, cancer, unintentional injuries, stroke, and homicide) are preventable [2]. Although many leading causes of premature death are preventable, research published in *The Lancet* using Centers for Disease Control and Prevention (CDC) data projected that Black men would continue to experience disproportionately high rates of premature mortality through 2030, particularly from heart disease, even as overall mortality rates were projected to decline [3]. Thus, it is clear that additional efforts are urgently needed to promote life expectancy among Black men by addressing preventable causes of disease.

Given the high rates of preventable health concerns among Black men have persisted, it is evident that “one-size-fits-all” health promotion efforts have had limited impact. That is because context matters when implementing behavioral change programs [4]. Black men face distinctive contextual and systemic barriers to health, such as anti-Black racism, justified medical mistrust, and unequal access to health promoting resources [5, 6]. This highlights the importance of partnering with Black men (i.e., community-based participatory research) to account for these contextual and systemic barriers into the design, and implementation of health promotion efforts for Black men [4]. In addition to these barriers, there are significant researcher-derived barriers to promoting health among and in partnership with Black men. Studies have documented how there has been limited engagement among academic researchers in working with Black men to promote their health due to researcher bias [7]. Together, these barriers serve as evidence that health promotion efforts should be specifically targeted for Black men, as effectiveness of health promotion efforts influencing health behaviors, and outcomes, are contingent on incorporation of their lived experiences and contextual realities [8, 9].

### Barbershops and Health Promotion

For many Black men in the U.S., barbershops serve as a culturally significant, and trusted space holding deep cultural meaning for Black men to foster safe community, connection, and conversation [10]. Although barbershops have been a place of health promotion for generations among Black men [11], their cultural importance and significance has only recently been recognized and leveraged by academic researchers to implement health promotion programming for Black men [12]. Such programming has targeted prostate cancer screening, hypertension, violence reduction, and HIV risk among other preventable health concerns; and evidence suggests that programming implemented in barbershops are often associated with positive health-related changes [12]. One mechanism as to why barbershop-based programs have been successful may stem from their environmental characteristics (e.g., an environment by and for Black men that allows for difficult conversations about health, race, and gender) that have been cultivated by Black men for many generations. Qualitative data collected among Black men at barbershops indicates that the supportive environment and social nature of the barbershop facilitates positive health-related changes among Black men [13].

### Barbers as Health Promotion Specialists

In addition to barbershops being places of health promotion for generations, barbers have also served as formal and informal health providers for men. The history of barbering is filled with examples; including, when access to physicians was limited or restricted, barbers were known to perform tooth extractions, cauterizations, and surgery due to their skills with razors and shears [14]. In the modern day, barbers continue to engage in health promotion efforts; for example, in the 1970s Dr. Elijah Saunders engaged barbers in Baltimore to screen and spread information about hypertension in Black communities [15, 16]. More recently, research has been conducted with barbers to understand their capacity to engage in similar health promotion efforts. One study found that most barbers (82.1%) reported interest in collaborating with universities and engaging in health outreach programs [17]. Another study found that 92% of barbers indicated that they wanted training in skin cancer awareness [18]. Finally, another study found that barbers working within barbershops had a high amount of organizational readiness to implement health promotion efforts [19]. Although there is a long history of health promotion efforts taking place in the barbershop, and motivation and readiness to implement health promotion programming among barbers is high, research with barbers has indicated that interventions implemented by barbers should fit within their normal work routine, recognizing their time constraints and the intensity of their work schedules [20].

### Assessing the Importance and Feasibility of Health Topics for Barbershop-Based Health Programming

Given the work demands of barbers, health promotion programming among Black men in barbershops should recognize health topics that can be readily implemented by barbers. Identifying health topics that are important among Black men and feasible to address in barbershops by barbers and clients [21, 22] is critical to the success of barbershop-based health promotion programming. Assessing direct input from potential implementers (i.e., barbers) and recipients (i.e., clients) is important to the success of health promotion programming as it ensures accurate considerations of Black men’s lived experiences, and contextual factors that shape the feasibility and importance of health topics. This is consistent with community-engaged research methods emphasizing the centrality of amplifying voices while promoting the social validity of a program [23]. Thus, the present study used a twostep modified Delphi process to identify health topics that were important among Black men and feasible to address in barbershops by barbers.

## Methods

### Participants

A total of 63 participants met eligibility criteria and were included in the present study. The sample consisted of 29 barbers and 34 barbershop clients recruited from barbershops that serve predominantly Black or African American male clientele across the state of South Carolina. Among barbers (n = 29), the majority identified as male (79%) and Black or African American (97%). Barbers ranged in age from 19 to 64, with a mean age of 39.7 years (SD = 11.8). More than half of the barbers reported their marital status as single (62%), followed by married or in a domestic partnership (35%). Regarding barber employment status, most barbers reported being self-employed (66%) and/or owner of a barbershop (28%). Barbers’ annual income varied greatly, with over a third (35%) of barbers reporting an annual income between $40,000–59,000, followed by less than $20,000 (24%), and $60,000–79,000 (24%). Additionally, barbers reported a mean of 15.7 years (SD = 12.4) of experience as a barber and a mean of 8.1 years (SD = 9.0) working at their current barbershop. On average, barbers reported working 5.8 days a week (SD = 2.4), seeing 11.8 (SD = 11.1) clients per day, with appointments lasting 39.8 (SD = 20.5) minutes on average.

Among the barbershop clients (n = 34), all identified as Black or African American males (100%). Clients ranged in age from 19 to 64, with a mean age of 38.7 years (SD = 12.4). More than half of the clients reported their marital status as single (56%), followed by married or in a domestic relationship (38%). Most clients reported their employment status as full-time (84%). Annual income varied, with over a third of clients reporting annual incomes of $60,000–79,000 (39%), followed by $40,000–59,000 (24%), and $20,000–39,000 (15%). Additionally, clients reported receiving services from their current regular barber between less than 1 year to 24 years, with an average of 8.3 years. Clients also reported that the average appointment duration is 26.2 (SD = 12.6) minutes and the average length of time spent at the barbershop (not including time spent receiving a service) is 48.7 (SD = 29.0) minutes (e.g., time spent in the waiting room and/or conversing with other clients and barbers). Finally, clients were asked how well their barber knows them on a scale of 1 (not very well) to 10 (very well) and how comfortable they feel talking to their barber about health-related topics on a scale of 1 (not very comfortable) to 10 (very comfortable). Most clients (80%) reported a score of 7 or higher regarding how well their barber knows them as well as how comfortable they feel talking to their barber about health related topics. See Table 1 for additional participant demographic information.

**Table 1:** Demographic characteristics of participants, N = 63

Characteristic	Total (N = 63)		Barbers (n = 29)		Clients (n = 34)	
	n	%	n	%	n	%
**Sex**						
Male	57	90	23	79	34	100
Female	4	6	4	14	0	0
Non-binary	0	0	–	–	0	0
Prefer not to say	1	2	1	3	0	0
Prefer to self-describe	1	2	1	3	0	0
Race/Ethnicity*						
Black or African American	63	98	28	97	34	100
Hispanic or Latino	0	0	0	0	0	0
Caucasian	1	2	1	3	0	0
Asian	0	0	0	0	0	0
Native American or Alaskan Native	0	0	0	0	0	0
Native Hawaiian or Pacific Islander	0	0	0	0	0	0
Prefer to self-describe	0	0	0	0	0	0
**Marital Status**						
Single	37	59	18	62	19	56
Married/Domestic Partnership	23	37	10	35	13	38
Divorced	1	2	1	3	0	0
Widowed	0	0	0	0	0	0
Separated	2	3	0	0	2	6
Barbershop Employment*						
Owner	–	–	8	28	–	–
Employee	–	–	1	3	–	–
Self-employed	–	–	19	66	–	–
Apprentice/Assistant	–	–	5	17	–	–
Renting a Chair	–	–	2	7	–	–
Other	–	–	3	10	–	–
**Employment Status**						
Full-time	–	–	–	–	27	84
Part-time	–	–	–	–	3	9
Retired	–	–	–	–	0	0
Other	–	–	–	–	2	6
**Annual Income**						
Less than $20,000	10	17	7	24	3	9
$20,000–39,000	6	10	1	3	5	15
$40,000–59,000	18	30	10	35	8	24
$60,000–79,000	20	33	7	24	13	39
$80,000–99,000	4	7	1	3	3	9
$100,000 and above	2	3	1	3	1	3

*Select all that apply – percentages may be greater than 100; Note: 2 barbers and 1 client did not report demographic information

### Procedures

The surveys were administered online via Qualtrics after approval was received from the University of South Carolina’s Institutional Review Board. Eligibility criteria for the Round 1 barber survey were that participants (1) were barbers in the state of South Carolina, (2) age 18 or older and, (3) worked with predominantly Black or African American male clientele. Convenience and snowball sampling procedures were used. First, barbers were recruited from the Clean Cuts and Sharp Minds Collective (CCSMC)—an existing network of South Carolina barbershops that serve predominantly Black male clientele. Members of the research team also recruited barbers by visiting local barbershops in person and requesting referrals from current eligible barbers.

Eligibility criteria for Round 2 client survey were that participants (1) identified as Black or African American males, (2) were age 18 or older, (3) resided in the state of South Carolina, and (4) were a current or former client at a barbershop in South Carolina. Following Round 1 survey data collection with barbers, Black or African American male barbershop clients for Round 2 survey were recruited from barbershops that participated in Round 1 of the survey. The inclusion criteria for Round 1 barber survey were assessed and verified through verbal confirmation with a member of the research team and through self-report on a prescreening survey for Round 2 client survey. Eligible participants were provided a brief description of the study at the start of the survey, informing them of the estimated survey completion time of 20 minutes and the compensation rate of a $20 Visa gift card (sent via mail) for completing the survey.

### Measures

#### Round 1: Barbers

Round 1 of the Delphi survey was administered to barbers. Informed by prior qualitative studies with Black men in South Carolina [24], common health concerns listed by the CDC, and feedback from the CCSMC, the survey was comprised of a comprehensive list of 54 different health topics related to physical, social, mental, and spiritual health . Barbers were prompted to rate (on a Likert scale of 1 = “not at all” to 5 = “extremely”) both the importance and feasibility (i.e., how realistic) of addressing that health topic among their Black male clientele in the barbershop. After rating the importance and feasibility of each health topic, barbers were given the option to add additional health topics. The survey concluded with demographic and background questions (e.g., race, age, number of years experience as a barber). A sample item from the Round 1 barber survey is “Please indicate how important it would be to address safe sex practices as a health topic with Black male customers in your barbershop,” and “Please indicate how realistic it would be to address safe sex practices as a health topic with Black male customers in your barbershop.”

**Table 2:** Round 1: barber ratings of health topic importance and feasibility, n = 29

#	Health topic	Importance			Feasibility			Go zone	Round 2 decision
		*M*	SD	Range	*M*	SD	Range		
12	Suicide	4.59	0.87	1–5	3.83	1.28	1–5	1	Retained
43	Safe sex practices	4.52	0.79	1–5	4.38	0.94	2–5	1	Retained
47	Community violence/safety	4.52	0.91	1–5	4.41	0.78	3–5	1	Retained
54	Parenting	4.52	0.74	2–5	4.28	0.84	2–5	1	Retained
16	High blood pressure	4.48	0.74	2–5	4.28	1.03	2–5	1	Retained
49	Social support	4.45	0.74	3–5	4.14	0.92	2–5	1	Retained
51	Child health	4.45	0.74	2–5	4.21	0.86	2–5	1	Retained
28	Nutrition	4.41	0.79	2–5	4.21	0.82	3–5	1	Retained
13	Mental Health Service Use/Therapy	4.38	0.86	2–5	4.00	1.17	2–5	1	Retained
46	Discrimination	4.38	1.05	1–5	4.10	1.18	1–5	1	Retained
52	Family relationships/ dynamics	4.38	0.78	2–5	4.21	0.94	2–5	1	Retained
1	Anxiety	4.34	0.67	3–5	3.86	0.99	2–5	1	Retained
17	Diabetes	4.34	0.81	2–5	4.14	0.95	2–5	1	Retained
18	Heart disease	4.34	0.81	2–5	3.97	1.02	2–5	1	Retained
29	Exercise	4.34	0.81	2–5	4.28	0.80	2–5	1	Retained
45	Institutional racism	4.34	1.01	1–5	4.07	1.13	1–5	1	Retained
20	Stroke	4.31	0.85	2–5	4.1	1.15	1–5	1	Retained
5	Depression	4.28	0.88	1–5	3.97	1.09	1–5	1	Retained
9	Anger	4.28	0.80	2–5	4.03	0.87	2–5	1	Retained
10	Trauma	4.28	0.75	3–5	3.93	1.03	2–5	1	Retained
14	Physical Health Care Use	4.24	0.74	2–5	3.86	0.95	2–5	1	Retained
24	Prostate cancer	4.24	1.15	1–5	3.90	1.32	1–5	1	Retained
39	HIV/AIDS	4.24	1.09	1–5	3.86	1.30	1–5	1	Retained
8	Recreational drug use	4.21	0.86	2–5	4.00	1.00	2–5	1	Retained
30	Weight	4.21	0.81	2–5	4.07	0.96	2–5	1	Retained
2	Work-related stress	4.17	1.07	1–5	4.03	1.09	1–5	1	Retained
6	Alcohol use	4.10	1.01	2–5	3.93	1.10	2–5	1	Retained
3	Family-related stress	3.93	1.07	1–5	3.76	1.02	1–5	2	Retained
4	Money-related stress	3.93	0.88	2–5	3.86	0.92	2–5	2	Retained
31	Sleep	3.97	1.18	1–5	3.48	1.33	1–5	3	Removed
35	Chlamydia	3.9	1.21	1–5	3.41	1.30	1–5	3	Removed
38	Herpes	3.9	1.37	1–5	3.62	1.43	1–5	3	Removed
26	Skin cancer	3.79	1.26	1–5	3.24	1.41	1–5	3	Removed
36	Gonorrhea	3.79	1.21	1–5	3.48	1.33	1–5	3	Removed
42	Hepatitis	3.76	1.27	1–5	3.52	1.3	1–5	3	Removed
53	Family planning/reproductive health	3.76	1.06	1–5	3.41	1.27	1–5	3	Removed
7	Tobacco use	3.72	1.28	1–5	3.69	1.31	1–5	3	Removed
11	Loneliness	3.72	1.31	1–5	3.38	1.18	1–5	3	Removed
40	HPV	3.72	1.28	1–5	3.28	1.39	1–5	3	Removed
37	Syphilis	3.69	1.37	1–5	3.24	1.48	1–5	3	Removed
32	Erectile dysfunction	3.55	1.24	1–5	2.86	1.30	1–5	3	Removed
50	Aging	3.55	1.15	1–5	3.62	1.27	1–5	3	Removed
48	Religion/spirituality	3.48	1.15	1–5	3.34	1.31	1–5	3	Removed
21	COVID-19	3.45	1.33	1–5	3.62	1.18	1–5	3	Removed
44	Same sex relationships	3.38	1.45	1–5	3.00	1.36	1–5	3	Removed
41	Genital warts	3.24	1.48	1–5	2.62	1.4	1–5	3	Removed
22	Flu	3.21	1.35	1–5	3.00	1.28	1–5	3	Removed
15	Asthma	3.17	1.28	1–5	3.07	1.16	1–5	3	Removed
34	Premature ejaculation	3.00	1.41	1–5	2.62	1.35	1–5	3	Removed
33	Low sex drive	2.93	1.19	1–5	2.90	1.21	1–5	3	Removed
19	High cholesterol	4.17	0.89	2–5	3.62	1.21	1–5	4	Collapsed w/ 28
23	Lung Cancer	4.03	1.02	1–5	3.66	1.32	1–5	4	Retained
27	Liver cancer	4.03	0.94	1–5	3.59	1.21	1–5	4	Retained
25	Colorectal cancer	4.00	1.13	1–5	3.55	1.35	1–5	4	Retained

#### Round 2: Clients

Round 2 of the Delphi survey was administered to Black male barbershop clients. Clients were also prompted to rate the importance and feasibility (on a Likert scale of 1 = “not at all” to 5 = “extremely”) of barbers addressing each health topic with their Black male clients at their barbershop. Consistent with the Delphi method, the list of health topics included in the Round 2 client survey was updated based on Round 1 barber results. Specifically, the list of health topics was reduced from 54 in the Round 1 barber survey to 32 in Round 2 client survey. Health topics deemed by barbers as the least important and feasible (i.e., Go-Zone 3 ) to address with their Black male clients in the barbershop were the health topics that were removed from Round 2 client survey. After rating the importance and feasibility of each health topic, clients were also given the option to add additional health topics. The survey concluded with demographic and background questions (e.g., race, age, number of years seeing current barber). Sample items from Round 2 client survey include “Please indicate how important it would be for barbers to address safe sex practices as a health topic with their Black male customers at their barbershop,” and “Please indicate how realistic it would be for barbers to address safe sex practices as a health topic with their Black male customers at their barbershop.”

**Fig. 1:**
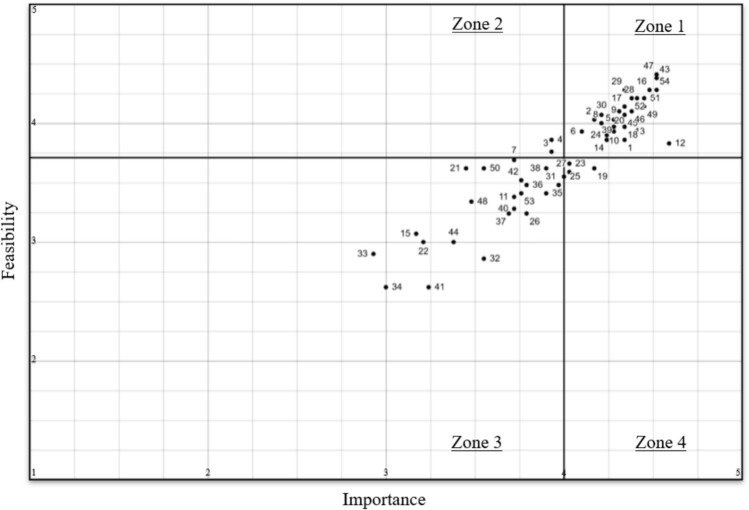
Round 1 Go-Zone Plot: Barber Ratings, n = 29

### Data Analyses

Descriptive analyses of quantitative importance and feasibility ratings were conducted using SPSS Version 30.0. Mean importance and feasibility ratings of each health topic were plotted on a Go-Zone plot to compare ratings within quadrants. The quadrants are divided by the grand mean ratings of importance and feasibility. The quadrants facilitate visual comparisons of health topics’ by importance and feasibility ratings, with health topics falling in Go-Zone 1 representing the health topics rated highly on both importance and feasibility, Go-Zone 2 representing the health topics rated low on importance and high on feasibility, Go-Zone 3 representing health topics rated low on importance and feasibility, and Go-Zone 4 representing health topics rated high on importance and low on feasibility , , . Independent samples *t*-tests were also conducted to infer statistically significant differences between barber and client ratings of the importance and feasibility of addressing various health topics in the barbershop. Paired samples *t*-tests were conducted to infer statistically significant differences between importance and feasibility ratings within each group (i.e., barbers and clients).

**Fig. 2:**
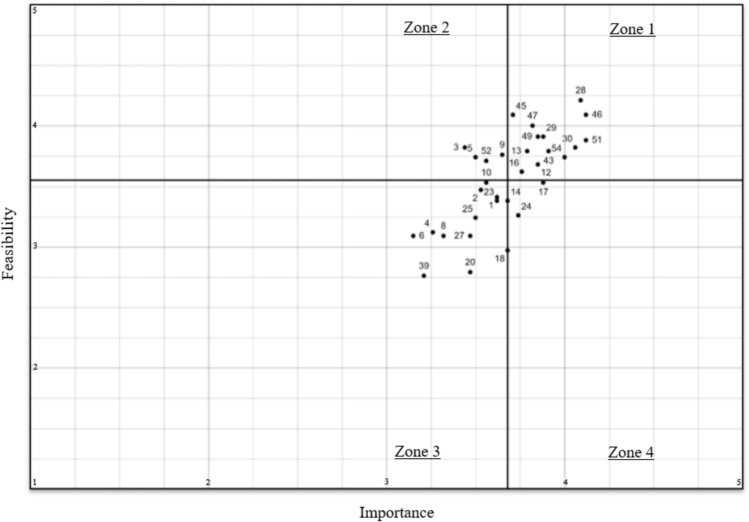
Round 2 Go-Zone Plot: Client Ratings, n = 34

**Fig. 3:**
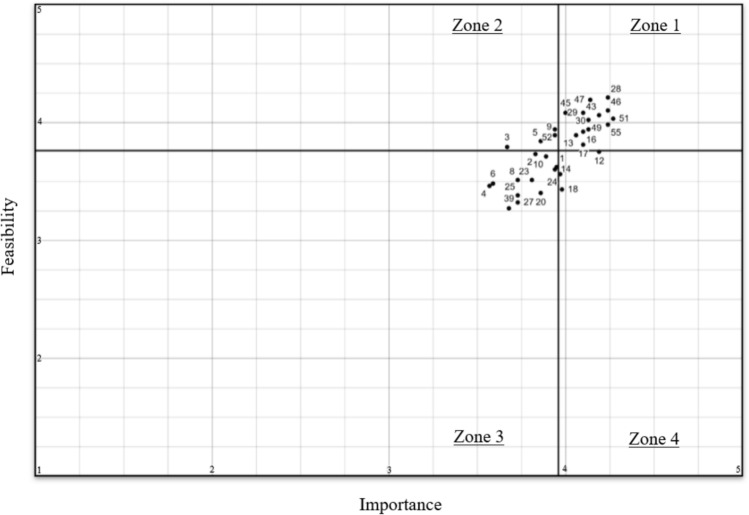
Go-Zone Plot: Barber and Client Ratings Combined, N = 63

To reduce the list of health topics from Round 1 barber survey to Round 2 client survey, an Excel spreadsheet was used to display mean ratings for each health topic, the respective Go-Zone number, as well as qualitative data from optional open-ended survey questions representing barbers’ additional comments and considerations of each health topic. Authors determined to remove health topics in Go-Zone 3 (i.e., health topics deemed by barbers as the least important and feasible to address with their clients). This decision was informed by literature emphasizing implementers’ perceived acceptability and feasibility as key determinants of intervention adoption. In other words, if barbers do not believe a health topic is important or feasible to address in the barbershop, they may be less likely to adopt the intervention [25]; thus, clients were not administered the health topics that barbers rated least important and feasible. Updates were also made to Round 2 client survey based on open-ended comments from Round 1 barber survey. Additional health topics listed by barbers were compiled in an Excel spreadsheet and reviewed/coded by the research team.

## Results

### Round 1 Barber Results

All 54 health topics received importance and feasibility ratings from barbers (n = 29). Most health topics (n = 32, 59%) were rated by barbers as important and/or feasible to address with their Black male clients in the barbershops. The 21 health topics that received low importance and feasibility ratings (i.e., Go-Zone 3) and were removed from Round 2 client surveys are as follows: tobacco use (7), loneliness (11), asthma (15), COVID-19 (21), flu (22), skin cancer (26), sleep (31), erectile dysfunction (32), low sex drive (33), premature ejaculation (34), chlamydia (35), gonorrhea (36), syphilis (37), herpes (38), HPV (40), genital warts (41), hepatitis (42), same sex relationships (44), religion/spirituality (48), aging (50), and family planning/reproductive health (53). Additionally, based on open-ended comments, one health topic was collapsed with another health topic. Specifically, barbers viewed high cholesterol as closely connected to nutrition; thus, high-cholesterol (19) was collapsed with the nutrition (28) health topic and not included in Round 2 client survey. The authors determined that all additional health topics suggested by barbers were already represented by the pre-existing list of health topics (e.g., “healthy eating” was listed as an additional health topic and is a synonym to “nutrition” that was included in the pre-existing health topic list). Thus, no new health topics suggested by barbers were added to Round 2 client survey. For Round 1 barber survey, importance ratings ranged from 2.93 (low sex drive) to 4.59 (suicide) and feasibility ratings ranged from 2.62 (premature ejaculation) to 4.41 (community violence/safety). See Table 2 and Fig. 1 for barber importance and feasibility ratings of the 54 health topics.

### Round 2 Client Results

Clients (n = 34) were administered the remaining 32 health topics based on Survey 1 barber results. All health topics received importance and feasibility ratings from clients. Most health topics (n = 21, 66%) were rated by clients as important and/or feasible for barbers to address with their Black male clients in the barbershop. The health topics that received low importance and feasibility ratings (i.e., Go-Zone 3) from clients are as follows: anxiety (1), work-related stress (2), money-related stress (4), alcohol use (6), recreational drug use (8), trauma (10), stroke (20), lung cancer (23), colorectal cancer (25), liver cancer (3), and HIV/AIDS (39). Importance ratings ranged from 3.15 (alcohol use) to 4.12 (children’s health and discrimination) and feasibility ratings ranged from 2.76 (HIV/AIDS) to 4.21 (nutrition). See Table 3and Fig. 2 for client importance and feasibility ratings of the 32 health topics.

**Table 3:** Round 2: client ratings of health topic importance and feasibility, n = 34

#	Health Topic	Importance			Feasibility			Go-Zone
		*M*	SD	Range	*M*	SD	Range	
46	Discrimination	4.12	1.07	2–5	4.09	1.11	1–5	1
51	Child health	4.12	1.07	2–5	3.88	1.23	1–5	1
28	Nutrition	4.09	1.14	2–5	4.21	1.12	1–5	1
30	Weight	4.06	1.3	1–5	3.82	1.36	1–5	1
54	Parenting	4.00	1.13	2–5	3.74	1.29	1–5	1
43	Safe sex practices	3.91	1.14	2–5	3.79	1.3	1–5	1
29	Exercise	3.88	1.27	1–5	3.91	1.16	1–5	1
12	Suicide	3.85	1.44	1–5	3.68	1.43	1–5	1
49	Social support	3.85	1.31	1–5	3.91	1.24	1–5	1
47	Community violence/safety	3.82	1.17	1–5	4.00	1.10	2–5	1
13	Mental Health Service Use/Therapy	3.79	1.34	1–5	3.79	1.45	1–5	1
16	High blood pressure	3.76	1.21	1–5	3.62	1.37	1–5	1
45	Institutional racism	3.71	1.36	1–5	4.09	1.06	2–5	1
9	Anger	3.65	1.35	1–5	3.76	1.28	1–5	2
52	Family relationships/dynamics	3.56	1.13	1–5	3.71	1.27	1–5	2
5	Depression	3.50	1.40	1–5	3.74	1.42	1–5	2
3	Family-related stress	3.44	1.35	1–5	3.82	1.22	1–5	2
1	Anxiety	3.62	1.23	1–5	3.41	1.35	1–5	3
23	Lung Cancer	3.62	1.26	1–5	3.38	1.54	1–5	3
10	Trauma	3.56	1.26	1–5	3.53	1.33	1–5	3
2	Work-related stress	3.53	1.35	1–5	3.47	1.26	1–5	3
25	Colorectal cancer	3.50	1.52	1–5	3.24	1.63	1–5	3
20	Stroke	3.47	1.56	1–5	2.79	1.57	1–5	3
27	Liver cancer	3.47	1.33	1–5	3.09	1.53	1–5	3
8	Recreational drug use	3.32	1.39	1–5	3.09	1.62	1–5	3
4	Money-related stress	3.26	1.48	1–5	3.12	1.47	1–5	3
39	HIV/AIDS	3.21	1.61	1–5	2.76	1.42	1–5	3
6	Alcohol use	3.15	1.33	1–5	3.09	1.58	1–5	3
17	Diabetes	3.88	1.27	1–5	3.53	1.54	1–5	4
24	Prostate cancer	3.74	1.38	1–5	3.26	1.52	1–5	4
14	Physical Health Care Service Use	3.68	1.49	1–5	3.38	1.56	1–5	4
18	Heart disease	3.68	1.55	1–5	2.97	1.59	1–5	4

### Combined Results

Collectively, barbers and clients (N = 63) rated nearly half of the 32 health topics as important and/or feasible as represented by Go-Zones 1, 2, and 4 (n = 14, 44%). The health topics rated most important and feasible (i.e., Go-Zone 1) by both barbers and clients are as follows: mental health service use/therapy (13), high blood pressure (16), diabetes (17), nutrition (28), exercise (29), weight (30), safe sex practices (43), institutional racism (45), discrimination (46), community safety/violence (47), social support (49), children’s health (51), family relationships/dynamics (52), and parenting (54). Importance ratings ranged from 3.57 (money-related issues) to 4.27 (children’s health) and feasibility ratings ranged from 3.27 (HIV/AIDS) to 4.21 (nutrition). See Table 4 and Fig. 3 for combined barber and client importance and feasibility ratings of the 32 health topics.

**Table 4:** Barber and client combined ratings of health topic importance and feasibility, N = 63

#	Health Topic	Importance			Feasibility			Go-Zone
		*M*	SD	Range	*M*	SD	Range	
51	Child health	4.27	0.94	2–5	4.03	1.10	1–5	1
28	Nutrition	4.24	0.98	2–5	4.21	0.99	1–5	1
46	Discrimination	4.24	1.06	1–5	4.1	1.13	1–5	1
54	Parenting	4.24	1.00	2–5	3.98	1.13	1–5	1
43	Safe sex practices	4.19	1.03	2–5	4.06	1.18	1–5	1
47	Community violence/safety	4.14	1.11	1–5	4.19	0.98	2–5	1
30	Weight	4.13	1.14	1–5	3.94	1.19	1–5	1
49	Social support	4.13	1.11	1–5	4.02	1.1	1–5	1
16	High blood pressure	4.10	1.07	1–5	3.92	1.26	1–5	1
17	Diabetes	4.10	1.10	1–5	3.81	1.33	1–5	1
29	Exercise	4.10	1.10	1–5	4.08	1.02	1–5	1
13	Mental Health Service Use/Therapy	4.06	1.18	1–5	3.89	1.32	1–5	1
45	Institutional racism	4.00	1.24	1–5	4.08	1.08	1–5	1
52	Family relationships/dynamics	3.94	1.06	1–5	3.94	1.15	1–5	1
9	Anger	3.94	1.16	1–5	3.89	1.11	1–5	2
5	Depression	3.86	1.24	1–5	3.84	1.27	1–5	2
3	Family-related stress	3.67	1.21	1–5	3.79	1.12	1–5	2
1	Anxiety	3.95	1.07	1–5	3.62	1.21	1–5	3
10	Trauma	3.89	1.11	1–5	3.71	1.21	1–5	3
20	Stroke	3.86	1.34	1–5	3.4	1.53	1–5	3
2	Work-related stress	3.83	1.26	1–5	3.73	1.21	1–5	3
23	Lung Cancer	3.81	1.16	1–5	3.51	1.45	1–5	3
8	Recreational drug use	3.73	1.25	1–5	3.51	1.44	1–5	3
25	Colorectal cancer	3.73	1.37	1–5	3.38	1.51	1–5	3
27	Liver cancer	3.73	1.19	1–5	3.32	1.4	1–5	3
39	HIV/AIDS	3.68	1.48	1–5	3.27	1.46	1–5	3
6	Alcohol use	3.59	1.28	1–5	3.48	1.44	1–5	3
4	Money-related stress	3.57	1.28	1–5	3.46	1.29	1–5	3
12	Suicide	4.19	1.26	1–5	3.75	1.36	1–5	4
18	Heart disease	3.98	1.3	1–5	3.43	1.43	1–5	4
24	Prostate cancer	3.97	1.30	1–5	3.56	1.46	1–5	4
14	Physical Health Care Service Use	3.94	1.23	1–5	3.6	1.33	1–5	4

### Barber and Client Comparisons

An independent samples* t*-test revealed that barbers (*M* = 4.29, *SD* = 0.52) reported statistically significant higher importance ratings than clients (*M* = 3.68, *SD* = 0.63), *t*(61) = 4.16,* p* < .001, *d* = 1.05. A second independent samples* t*-test also revealed that barbers (*M* = 4.01, *SD* = 0.67) report statistically significant higher feasibility ratings than clients (*M* = 3.55, *SD* = 0.64), *t*(61) = 2.80,* p* = .007, *d* = 0.71. See Table 5 for independent samples *t*-test results.

**Table 5:** Independent samples T-Test, N = 63

	Barbers		Clients		*t*(61)	*p*	Cohen’s* d*
	*M*	*SD*	*M*	*SD*			
Importance	4.29	0.52	3.68	0.63	4.16	< .001	1.05
Feasibility	4.01	0.67	3.55	0.64	2.80	.007	0.71

A paired-samples *t*-test revealed that importance ratings (*M* = 4.29, *SD* = 0.52) were rated statistically significantly higher than feasibility ratings (*M* = 4.01, *SD* = 0.67) by barbers, *t*(61) = 3.01,* p* = .005, *d* = 0.56. A paired-samples *t*-test revealed that there were no statistically significant differences between importance (M = 3.68, *SD* = 0.63) and feasibility (*M* = 3.55, *SD* = 0.64) ratings of clients, *t*(61) = 1.72,* p* = .10, *d* = 0.29. See Table 6 for paired-samples *t*-test results.

**Table 6:** Paired-samples T-Test, N = 63

	Importance		Feasibility		*t*(61)	*p*	Cohen’s *d*
	*M*	*SD*	*M*	*SD*			
Barbers	4.29	0.52	4.01	0.67	3.01	.005	0.56
Clients	3.68	0.63	3.55	0.64	1.72	.10	0.29

## Discussion

Innovative efforts to promote health and life expectancy among Black men are needed. The current study can inform these efforts because it identifies health topics that are important among Black men and feasible to address in the barbershop setting. Barbershops serve as important cultural institutions for Black men, fostering connection and community. Additionally, barbers often function as change agents who play a critical role in spreading health information and supporting health promotion efforts for Black men in their community. However, given barbers’ high workload, it is important to identify health topics that are important among Black men and feasible for barbers to address among their Black male clients, to ensure effectiveness, and sustainability of barbershop-based health promotion efforts for Black men.

Data from the current study indicated that there was a wide variety of health topics rated by barbers and clients as both important and feasible for barbers to address among Black men in the barbershop. These topics include: mental health service use/therapy, high blood pressure, diabetes, nutrition, exercise, weight, safe sex practices, institutional racism, discrimination, community safety/violence, social support, children’s health, family relationships/dynamics, and parenting. These findings indicate that it is likely that there would be widespread support by barbers and clients for barbershop-based health promotion programming targeting these topics.

Several findings from this study are particularly noteworthy as they align with the current state of the literature while also revealing significant gaps. First, institutional racism, discrimination, and community safety/violence are identified as important and feasible health concerns among barbers and clients. This finding corresponds with the extensive body of literature on the detrimental impact of institutional racism, discrimination, and community violence on the mental, physical, and social health of Black Americans. Second, children’s health, family relationships/dynamics, and parenting are identified as important and feasible health topics among barbers and clients. This finding aligns with research indicating that Black men are invested in the health of children in their communities and identify their families and children as significant motivators for engaging in health promoting behaviors [24, 26, 27]. Third, it is noteworthy that heart disease, prostate cancer, and stroke are missing from this list given that they are significant health concerns among Black men and contributors to premature mortality among Black men [2]. This last noteworthy finding indicates that additional work is needed to further understand barbers and clients perceptions of these health topics and/or spread awareness of these health concerns in barbershop settings.

Compared to their clients, barbers generally viewed a broader range of health topics as more important and feasible to address within the barbershop context. This finding suggests that barbers feel that their barbershops are open to a wide range of conversations about health. This is important because barbers are gatekeepers to the barbershop environment. As business owners and employees, they often have the final say in what can, and cannot be discussed, in their place of business. Thus, their willingness to address a wide-range of health topics in their places of business is important given the risks of implementation failure, or the failure of a well-designed intervention due to implementation-related (e.g., implementers/participants not feeling comfortable) concerns [28]. Qualitative research has identified program fit as a potential major contributor to implementation failure among barbershop-based health promotion programs [29]. This finding also indicates that pre-program efforts are needed to build the readiness of Black male clients to discuss certain health topics in the barbershop. Such efforts must recognize that various factors (i.e., limited awareness, perceived appropriateness, fear or anxiety, discussion with other trusted sources, hypermasculinity) may discourage open discussion of sensitive health-related issues that may be important among Black men [30, 31]; although it is noteworthy that clients identified topics, such as institutional racism, family-related stress, depression, anger, and community violence/safety, as most feasible in the barbershop. These topics require vulnerability and comfort to discuss, which may suggest that clients are open and willing to engage further with barbers on various health concerns.

Findings from the current study should be considered in light of several limitations. First, participants, including barbers and clients, were all located in South Carolina, which may limit the generalizability of results to individuals across the U.S.. Focus groups conducted in barbershops in South Carolina have identified unique contributors to health among Black men [24]. Future research efforts should expand samples to include barbers and clients from diverse geographic regions across the U.S. Furthermore, although the health topic list included in the current study was comprehensive, it may not encompass all health topics considered important or feasible by Black men and barbers; although it should be noted that the current method allowed for participants to enter additional health topics. Future efforts should inquire about additional topics relevant to Black men from barbers and clientele. Third, the health topics provided to barbers and clients were broad and may be perceived as varying in importance or feasibility if broken down further into more specific subtopics (e.g., “HPV” versus “HPV prevention” or “HPV treatment”). Finally, this study did not examine contextual factors that may have influenced participant ratings. Future research should consider using mixed-methods approaches to further explore how varying contextual factors shape perceptions of the importance and feasibility of these health topics. Such work may provide insight into strategies for improving perceptions of the health topics that received the lowest ratings.

Despite such limitations, the current study possesses several strengths and contributes to the existing literature. First, the current study addresses an important gap by focusing on health promotion among Black men, given high rates of preventable diseases and lower life expectancies of Black men relative to other racial and ethnic groups. Scoping reviews have found too few health promotion programs tailored for Black men [32], including those conducted in barbershops [12]. There still remains a significant gap in the literature on how to develop and implement such programming. Second, the current study recognizes the limited capacity of barbers given their high workload and intense job demands, which include maintaining interpersonal responsibilities with clientele. Programming implemented in barbershops must recognize that barbers have a primary job (i.e., barbering) and that programming should not impact their ability to make a livelihood. Given this, the study prioritizes health topics that are important and feasible in order to reduce barber burden, while still promoting meaningful health changes among Black men. Finally, the current study amplifies the voices of Black men centering them as change agents in their own health promotion efforts, ensuring intentional and meaningful engagement with what Black men identify as important and feasible. This ensures that health topics identified are related to the lived experiences of Black men.

Findings from the current study have important implications for the design and implementation of health promotion programming in barbershops for Black men. When designing and implementing health promotion efforts in barbershops, researchers must consider the health topics that can be readily addressed by barbers, given their demanding workload, and deemed important by both barbers and Black male clients. Programming addressing such topics can ensure accurate considerations of Black men’s lived experiences and contextual factors, helping to promote health equity, increase life expectancy, and improve health outcomes among and in partnership with Black men.
